# A Simple Criterion for Inferring CRISPR Array Direction

**DOI:** 10.3389/fmicb.2019.02054

**Published:** 2019-09-04

**Authors:** Ognjen Milicevic, Jelena Repac, Bojan Bozic, Magdalena Djordjevic, Marko Djordjevic

**Affiliations:** ^1^School of Medicine, University of Belgrade, Belgrade, Serbia; ^2^Multidisciplinary Ph.D. Program in Biophysics, University of Belgrade, Belgrade, Serbia; ^3^Faculty of Biology, Institute of Physiology and Biochemistry, University of Belgrade, Belgrade, Serbia; ^4^Institute of Physics Belgrade, University of Belgrade, Belgrade, Serbia

**Keywords:** CRISPR/Cas, non-canonical functions, CRISPR array orientation, large-scale analysis, *cas* gene orientation

## Abstract

Inferring transcriptional direction (orientation) of the CRISPR array is essential for many applications, including systematically investigating non-canonical CRISPR/Cas functions. The standard method, CRISPRDirection (embedded within CRISPRCasFinder), fails to predict the orientation (ND predictions) for ∼37% of the classified CRISPR arrays (>2200 loci); this goes up to >70% for the II-B subtype where non-canonical functions were first experimentally discovered. Alternatively, Potential Orientation (also embedded within CRISPRCasFinder), has a much smaller frequency of ND predictions but might have significantly lower accuracy. We propose a novel simple criterion, where the CRISPR array direction is assigned according to the direction of its associated *cas* genes (Cas Orientation). We systematically assess the performance of the three methods (Cas Orientation, CRISPRDirection, and Potential Orientation) across all CRISPR/Cas subtypes, by a mutual crosscheck of their predictions, and by comparing them to the experimental dataset. Interestingly, CRISPRDirection agrees much better with Cas Orientation than with Potential Orientation, despite CRISPRDirection and Potential Orientation being mutually related – Potential Orientation corresponding to one of six (heterogeneous) predictors employed by CRISPRDirection – and being unrelated to Cas Orientation. We find that Cas Orientation has much higher accuracy compared to Potential Orientation and comparable accuracy to CRISPRDirection – while accurately assigning an orientation to ∼95% of the CRISPR arrays that are non-determined by CRISPRDirection. Cas Orientation is, at the same time, simple to employ, requiring only (routine for prokaryotes) the prediction of the associated protein coding gene direction.

## Introduction

Clustered Regularly Interspaced Short Palindromic Repeats (CRISPR) arrays and associated Cas (CRISPR-associated) proteins constitute an adaptive prokaryotic immune system. It is considered that the system’s main role is to protect the cell from foreign DNA attack (bacteriophage or plasmid DNA) (Brouns et al., 2008). CRISPR/Cas system can also regulate endogenous genes, and affect processes such as DNA repair, sporulation, antimicrobial resistance, virulence, etc. (Babu et al., 2011; Gunderson et al., 2015; Rajagopalan and Kroos, 2017; Shabbir et al., 2018; Heidrich et al., 2019; Wei et al., 2019). A subtype II-B CRISPR/Cas system encoded by *Francisella novicida*, was found to facilitate the infection propagation, which provided the first direct experimental evidence of non-canonical CRISPR/Cas functions (Sampson et al., 2013, 2019). Experimental evidence that CRISPR/Cas systems that belong to other subtypes (e.g., Type II-C, Type I-F), are also exhibiting non-canonical functions through different functional/mechanistic modalities, are now accumulating (Veesenmeyer et al., 2014; Li et al., 2016; Dugar et al., 2018). From the computational side, we recently provided evidence (Bozic et al., 2019) that Type I-E CRISPR/Cas system from *Escherichia coli* has a clear preference to target host bacterial sequences vs. more than 230 sequenced *E. coli* phages. The predicted distribution of crRNA targets in the host genome is highly non-random, with the preference to target transcriptionally active regions and dsDNA rather than mRNA sequences. This, together with more indirect evidence – that the content of the Type I-E CRISPR array in *E. coli* remained identical over significant evolutionary timescales (Savitskaya et al., 2017), and that the system is not activated even by virus infection (Patterson et al., 2017) – strongly suggests a dominantly non-canonical function of this classical CRISPR/Cas model system.

**FIGURE 1:**
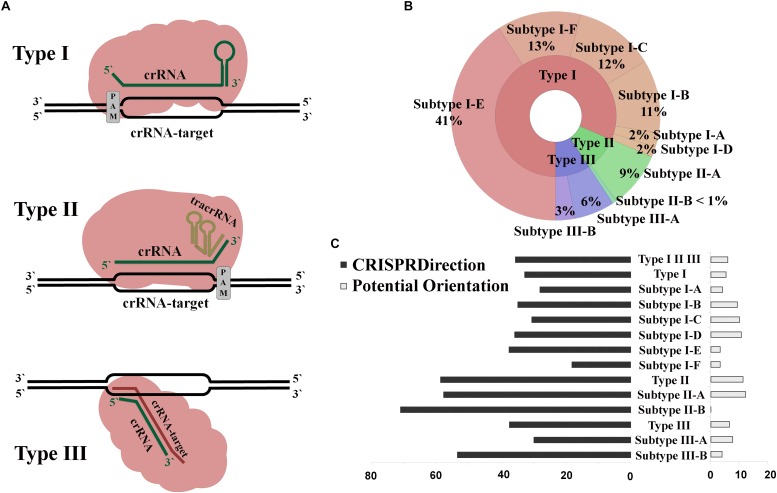
**(A)** Interference mechanism for CRISPR/Cas systems of Type I, II, and III. **(B)** Distribution of CRISPR/Cas loci throughout the domains of Bacteria and Archaea; **(C)** Percentage distribution of CRISPR arrays with non-defined orientation, by CRISPRDirection and Potential Orientation, across different CRISPR/Cas subtypes.

How widespread are these alternative CRISPR/Cas functions throughout bacterial and archaeal domains? To address this computationally, one has to systematically examine CRISPR spacer (i.e., the corresponding crRNAs) interactions with host genome sequences. Either dsDNA [as experimentally found in II-B system of *F. novicida* (Ratner et al., 2019), and also computationally predicted for I-E in *E. coli* (Bozic et al., 2019)], or mRNA [as in I-F and I-C systems from, respectively, *Pseudomonas aeruginosa* and *Campylobacter jejuni* (Li et al., 2016; Dugar et al., 2018)] can be targeted in CRISPR/Cas non-canonical functions. Moreover, in canonical functions, the system can also target either dsDNA (in Type I and II) or mRNA (in Type III), as schematically shown in Figure 1A. Therefore, knowing the array orientation allows assessing crRNA interactions with sense vs. antisense DNA strand, and consequently separating *bona fide* targets from false positives. Likewise, when prior knowledge of the nature of the CRISPR-target is missing, as for the *E. coli* I-E system that we recently analyzed (Bozic et al., 2019), the array orientation enables assigning the underlying regulatory modality (dsDNA vs. mRNA targeting). The information on the CRISPR array orientation is indispensable even when crRNA is not the mediator of non-canonical activities, as in II-B system of *F. novicida*, where a duplex of small accessory RNAs (scaRNA:tracrRNA, small CRISPR/Cas-associated RNA and trans-activating crRNA, respectively) binds the target. In our previous work (Guzina et al., 2018), we predicted scaRNA:tracrRNA hybrids in many Type II systems, which indicates that non-canonical functions might be widespread in this type. As tracrRNA is complementary with crRNA, the array orientation is needed for the small accessory RNA annotation, and subsequently, for the accurate target prediction. Finally, the spacers are sampled in the CRISPR array through adaptation process (Yosef et al., 2012), which exhibits asymmetry with respect to two DNA strands (Vorontsova et al., 2015), likely since the adaptation substrates are generated through (unidirectional) DNA replication machinery (Ivancic-Bace et al., 2015; Levy et al., 2015). Consequently, the array direction is also needed to understand the mechanism through which the spacers may be sampled when targeting the self-genome in non-canonical interactions.

The CRISPR array orientation is commonly predicted by the CRISPRDirection method (Biswas et al., 2014), which combines six different empirical predictors. The method is included in CRISPRCasFinder (Couvin et al., 2018), which is a widely used pipeline for *de novo* CRISPR/Cas prediction and typization. Another popular pipeline for the CRISPR/Cas detection, which also utilizes CRISPRDirection for predicting the array orientation, is CRISPRDetect (Biswas et al., 2016). CRISPRDirection provides a prediction when its parameters surpass certain thresholds, otherwise, no orientation is assigned to the array (ND predictions). When CRISPRDirection provides prediction, it is considered accurate, but the high frequency of ND assignments is its main disadvantage in the systematic analysis of non-canonical functions (and in other larger scale applications). To address this problem, a simplified Potential Orientation method was proposed within CRISPRCasFinder, which predicts the array orientation based on the AT richness of its leader region (one of CRISPRDirection predictors). While it is plausible that this decreases the frequency of ND assignments, there are a number of leaderless CRISPR/Cas systems (Alkhnbashi et al., 2016), so the accuracy of Potential Orientation becomes a question [as also suggested in Couvin et al. (2018)].

We here propose Cas Orientation, which is a simple novel criterion for determining the CRISPR array orientation. Cas Orientation assigns the array direction based on the direction of the associated *cas* genes. It is not *a priori* evident that the *cas* genes and the CRISPR array should have the same direction, i.e., this simple criterion is highly non-trivial: (i) the CRISPR array and the *cas* genes may be independently transcribed (Westra et al., 2010), so mechanistically they can easily have the opposite orientations, (ii) e.g., restriction-modification systems (another type of bacterial immune systems) are often organized in divergent architectures (Semenova et al., 2005), (iii) it is known that in Type II-C systems, the *cas* genes and the CRISPR array can be often oppositely oriented (Zhang et al., 2013), and opposite orientations have also been found in Type I-A systems (Garrett et al., 2011; Gudbergsdottir et al., 2011; Lintner et al., 2011; Mousaei et al., 2016; Rollie et al., 2017), (iv) *cas* gene orientation is not one of the predictors in CRISPRDirection.

We here perform a large-scale analysis on all currently available prokaryotic genomes (∼14000), to assess their accuracy and perform a crosscheck of CRISPRDirection, Potential Orientation and Cas Orientation. We also compare the accuracy of Cas Orientation and Potential Orientation on CRISPRDirection ND set. We show that Cas Orientation has high accuracy, i.e., much larger than Potential Orientation, and comparable to CRISPRDirection – while providing a prediction for any classified CRISPR array (i.e., evading large ND problem of CRISPRDirection), and being much simpler and more intuitive. We provide a performance analysis of all three methods within each CRISPR/Cas subtype individually (for the CRISPR/Cas subtype distribution, see Figure 1B). For CRISPRDirection and Potential Orientation such analysis was not done before, but is important, as differences in their performance across different CRISPR/Cas subtypes might be significant. For Cas Orientation, we show that its performance is particularly well suited to those subtypes involved in non-canonical functions, or where mRNA targeting may be exhibited (Types II and III).

## Materials and Methods

### Sequence Datasets and CRISPRCasFinder Analysis

Complete genome sequences of Bacteria and Archaea were retrieved from the NCBI assembly ftp site. The assemblies were downloaded using the NCBI Entrez python API on March 27, 2019, if they passed the filters “Complete genome” and “Has annotation” and, upon exclusion of plasmid sequences, further submitted to CRISPRCasFinder (standalone version – 4.2.17). Within CRISPRCasFinder, the parameters were set as follows: (i) “cas” was set to 1 (default is 0), so that *cas* genes are searched; (ii) “vicinity”, specifying the number of nucleotides separating the CRISPR array from the neighboring *cas* genes, was set to 1000 (default is 600), as somewhat larger distances during our analysis of Type II systems were noticed (Guzina et al., 2018); (iii) “rcfowce,” was set to 1 (default is 0) so that *cas* genes are searched only when a CRISPR array is found in the sequence; (iv) “definition,” specifying the stringency of the *cas* gene detection was set to “S,” so that the predicted CRISPR/Cas systems are subtyped based on the *cas* operon composition. The remaining parameters were set at their default values.

### Experimental Dataset Extension

The validation dataset, comprising a set of 25 repeats of experimentally determined orientation in 135 unique arrays, was gathered from reference (Biswas et al., 2014). This dataset was expanded to homologous arrays (with pre-assumed matching orientation, see section “Materials and Methods” in reference Biswas et al., 2014), by BLAST-ing repeat consensuses over the full set of NCBI prokaryotic genomes (downloaded April 2019) with the *E*-value cutoff of 10^–3^. Unique BLAST-ed genome sequences were further submitted to CRISPRCasFinder (under the parameters noted above) and for the predicted CRISPR/Cas systems, orientation from predictors of interest was obtained (CRISPRDirection, Potential Orientation, and Cas Orientation). The experimental information for this expanded dataset was assigned based on the original set information. This set was then divided to the loci where CRISPRDirection and Potential Orientation provide predictions (further called “Determined Orientation Set”) and where CRISPRDirection does not provide predictions (“ND Orientation Set”). Determined Orientation Set was then compared to all three methods, while ND Orientation Set was compared to Cas Orientation and Potential Orientation methods. For each analyzed predictor, the percentage of differing predictions (mismatch), with respect to the experimental orientation, was calculated.

To assign significance to the difference between two mismatching percentages, the following *P*-value calculation is consistently applied to all the results in the paper. Uncertainty for the mismatching counts is estimated based on the widely used assumption that the number of counts follows a Poisson distribution (i.e., corresponds to its standard deviation). Confidence intervals for the mismatching counts are then propagated to the mismatching percentages through standard uncertainty propagation (see e.g., Bevington and Robinson, 2002; Knezevic, 2008; Rouaud, 2013). The same uncertainty propagation procedure is also used to obtain confidence intervals for the difference between the mismatching percentages, from which *P*-values reported in the paper are calculated.

## Results and Discussion

### Prevalence and Orientation Assignment Bias of CRISPR/Cas Systems

Full set of complete bacterial and archaeal genomes (∼14000) was analyzed using the CRISPRCasFinder pipeline to infer a comprehensive list of CRISPR/Cas systems, which consist of independent CRISPR array and *cas* operon predictions. The CRISPR arrays labeled as Cas, Cas O, Cas U [described in detail in Couvin et al. (2018)] by CRISPRCasFinder were next excluded, as we further analyzed only classified CRISPR/Cas systems. Figure 1B shows a distribution of all analyzed CRISPR/Cas loci (5683 from 4353 genomes) over different subtypes (with ND categories from CRISPRDirection and Potential Orientation included). More than 80% of the CRISPR arrays belong to the Type I CRISPR/Cas systems, where the subtype I-E is the most prevalent. Even when the CRISPR loci with ND-orientation are excluded (leaving 3455 arrays in total), a similar distribution is observed (Supplementary Figure S1).

The array orientation which corresponds to CRISPRDirection, Potential Orientation, and Cas Orientation was then obtained from CRISPRCasFinder. As noted above, CRISPRDirection and Potential Orientation lead to ND assignments, which may present a serious limitation due to the lack of predictive power. Figure 1C shows the distribution of ND assignments for CRISPRDirection (left) and Potential Orientation (right) across different subtypes. Cas Orientation is not associated with ND category, as it predicts direction for every CRISPR/Cas system, due to the straightforward assignment of the direction for the associated *cas* genes.

Overall, CRISPRDirection fails to assign orientation for almost 40% of the analyzed CRISPR arrays (Supplementary Table S1). Moreover, the ND fractions in Figure 1C are non-uniformly distributed across different subtypes and are more pronounced for Types II and III (where determining the array direction may be particularly important, see section “Introduction”) compared to Type I. For example, for II-B subtype, where non-canonical functions were first experimentally discovered (Sampson et al., 2013), ND fraction is >70%. Potential Orientation leads to a significantly smaller ND fraction (∼6%), though its accuracy is also expected to be lower. However, this difference in accuracy was not quantified before and will be further assessed below, together with the accuracy of our newly proposed Cas Orientation.

### Mutual Comparison of CRISPRDirection, Cas Orientation, and Potential Orientation

**FIGURE 2:**
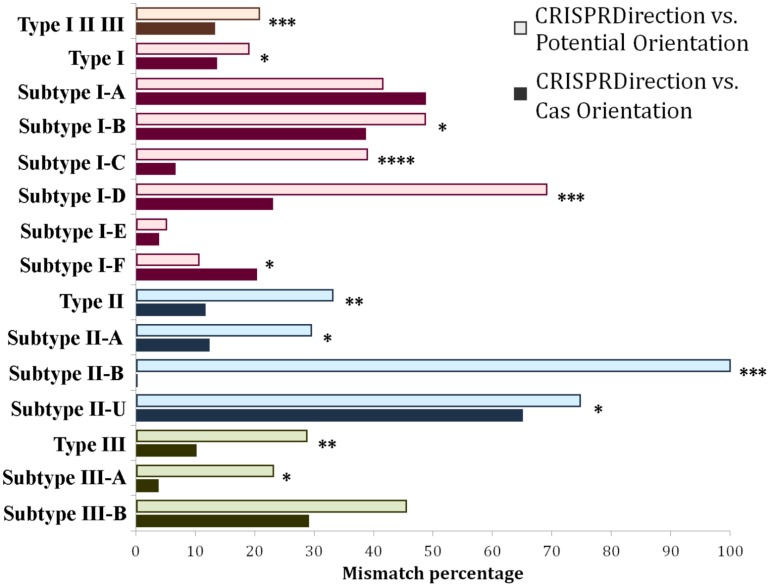
Distribution of mismatches between CRISPRDirection vs. Potential Orientation (lighter shade bar plots) as well as CRISPRDirection vs. Cas Orientation (darker shade bar plots) predictors across different CRISPR/Cas types and subtypes. Every CRISPR/Cas type (along with corresponding subtypes) was denoted with a different color, red corresponding to Type I, blue to Type II, and green to Type III systems. The entire dataset (Type I, Type II, and Type III) was denoted with an orange bar plot (^∗^*P* < 0.05; ^∗∗^*P* < 0.01; ^∗∗∗^*P* < 0.001; ^∗∗∗∗^*P* < 0.0001).

CRISPRDirection is widely considered to give accurate predictions of the CRISPR array orientation, with the problem that it leads to a high number of ND assignments (see above). Also generically, one may expect a better agreement of CRISPRDirection with Potential Orientation than with Cas Orientation, since Potential Orientation corresponds to one of the CRISPRDirection predictors, while CRISPRDirection does not use the *cas* gene orientation. Due to this, we start by mutually comparing CRISPRDirection predictions with Cas Orientation and Potential Orientation. Systematic comparison across the entire dataset (all three CRISPR/Cas Types), and across individual CRISPR/Cas subtypes is shown in Figure 2. Note that II-U is also included in the comparison, as it corresponds to the misclassified subtype II-C (as only core Type II *cas* genes are present in this subtype, i.e., no subtype specific genes are present).

Contrary to the generic expectation, Figure 2 shows that CRISPRDirection provides a better agreement with Cas Orientation than with Potential Orientation – the mismatching percentages at the entire dataset are 13% and 21%, respectively, which is statistically highly significant (P∼10^–4^) (Supplementary Table S2). The same trend is also observed across most of the individual subtypes. The only two exceptions are subtypes I-A and I-F, where Potential Orientation shows a better agreement with CRISPRDirection (not statistically significant for I-A). Differences between Potential Orientation and Cas Orientation agreements are pronounced for Types II and III, where the accurate orientation may be particularly important (see above), and where ND assignments by CRISPRDirection is large. For II-B subtype, which is a cornerstone for the non-canonical CRISPR/Cas paradigm (Sampson et al., 2013, 2019), CRISPRDirection has a perfect match with Cas Orientation and a complete mismatch with Potential Orientation. As an exception, for II-U/II-C there is a large mismatch of CRISPRDirection with Cas Orientation (and larger than with Potential Orientation). Overall, a better agreement of Cas Orientation with CRISPRDirection, which is contrary to the intuitive expectation, suggests that Cas Orientation may be an accurate (yet simple) predictor of CRISPR orientation, which moreover can assign an orientation to all classified CRISPR/Cas loci from ND set.

### Comparison With Experimental Dataset

**FIGURE 3:**
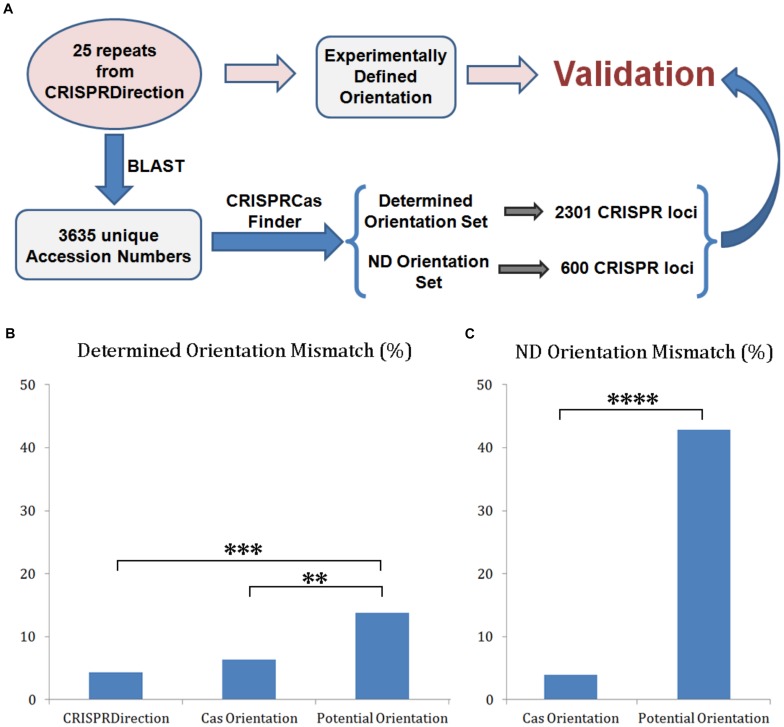
**(A)** Validation protocol for CRISPR array orientation predictors accuracy; **(B)** Distribution of mismatches between the investigated predictors (CRISPRDirection, Potential Orientation, and Cas Orientation) vs. Determined Orientation Set (^∗∗∗∗^*P* < 0.0001). **(C)** Distribution of mismatches between Potential Orientation and Cas Orientation vs. ND Orientation Set.

The experimental dataset was formed as described in the section “Materials and Methods,” and as schematically presented in Figure 3A (the blue labeled protocol). BLAST-ing 25 CRISPR repeats (from 135 unique arrays with the experimentally determined orientation) (Biswas et al., 2014) resulted in 3635 sequences with unique NCBI accession numbers. When submitted to CRISPRCasFinder, this resulted in the detection of 3015 classified CRISPR/Cas loci. The experimental orientation was propagated to this set based on the originating homologs from 25 CRISPR arrays dataset (Figure 3A, the red protocol). Determined Orientation Set was obtained by filtering-out those loci with ND orientation by either CRISPRDirection or Potential Orientation, which resulted in 2301 loci. ND Orientation Set was formed from those 600 loci with ND assignment by CRISPRDirection.

Determined Orientation Set was next compared to CRISPRDirection, Potential Orientation and Cas Orientation assignments, with the comparison presented as a mismatching percentage in Figure 3B. The mismatch percentage for Potential Orientation vs. Determined Orientation Set (14%) is higher compared to the percentages associated with Cas Orientation (6%) and CRISPRDirection (4%), where these differences are statistically significant at P∼10^–3^ and P∼10^–4^ levels, respectively (Supplementary Table S3). On the other hand, Cas Orientation and CRISPRDirection have comparable accuracy, with the corresponding difference not being statistically significant. Note here that CRISPRDirection is partially trained (parameterized) on the experimental dataset (Biswas et al., 2014), which to some extent increases its accuracy – no training (and parameterization) is needed for Cas Orientation.

The comparison in Figure 3B is done only for those CRISPR/Cas systems for which CRISPRDirection provides predictions, while (as a major advantage) Cas Orientation provides predictions on the entire set. It is consequently important to test the performance of the other two methods (Cas Orientation and Potential Orientation) for a large number of loci where CRISPRDirection leads to ND assignments (ND Orientation Set). This comparison is shown in Figure 3C, where mismatches of Cas Orientation and Potential Orientation with ND Orientation Set are shown. Strikingly, with respect to Figure 3B (comparison with Determined Orientation Set), the mismatches with Potential Orientation now increase by more than a factor of three (to 43%), while the mismatches with Cas Orientation even somewhat decrease (to ∼4%), leading to a notable statistical significance for the difference (P∼10^–14^) (Supplementary Table S4). For Potential Orientation, the large increase in mismatches is likely due to its relation to CRISPRDirection (see above) – i.e., where CRISPRDirection fails to provide predictions, Potential Orientation may also perform less well. So, to “resolve” CRISPRDirection ND assignments, a genuinely new predictor is needed, which is exactly what is provided by Cas Orientation.

**FIGURE 4:**
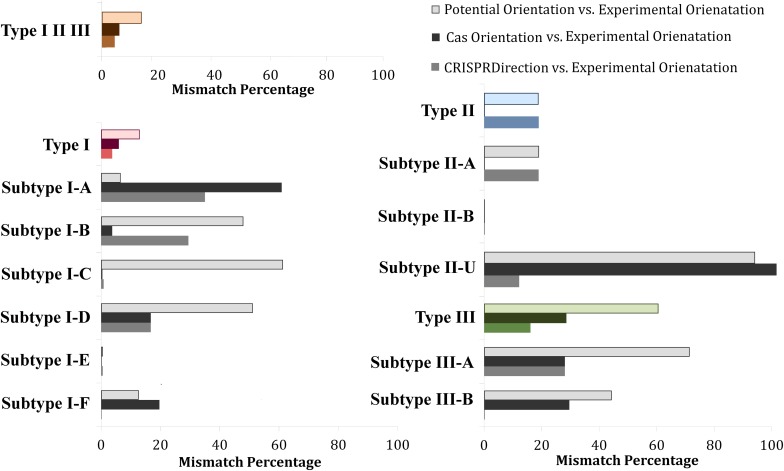
Distribution of mismatches between the investigated predictors (Potential Orientation, Cas Orientation, and CRISPRDirection) vs. Determined Orientation Set across different CRISPR/Cas types and subtypes. Every CRISPR/Cas type (along with corresponding subtypes) was denoted with a different color, red corresponding to Type I, blue to Type II, and green to Type III systems. The entire dataset (Type I, Type II, and Type III) was denoted with an orange bar plot. For each type, the gray-scale (printable) version of Potential Orientation (light shade), CRISPRDirection (moderate shade) and Cas Orientation (dark shade), correspond to the shades indicated in the figure legend. Note that II-U in the figure (labeled in accordance with CRISPRCasFinder notation), in fact corresponds to the subtype II-C.

Figure 4 shows mismatches for all three methods with respect to Determined Orientation Set, across individual CRISPR/Cas types and subtypes. The accuracy of CRISPRDirection is high on average (∼4% mismatches for a subset on which it provides predictions), but displays a notable heterogeneity across different subtypes – from (almost) perfect matches for I-E and I-F, to ∼40% mismatch for I-A and I-B. The high accuracy of CRISPRDirection and Potential Orientation for subtype I-E is expected – I-E has a notable representation in the experimental pool (∼25%) from which CRISPRDirection is in part trained, and a well-defined leader region (as relevant for Potential Orientation). For Cas Orientation, we observe comparable (or even somewhat better) accuracy to CRISPRDirection (I-B, I-C, I-D, I-E, II-A; II-U – to be discussed below); e.g., for Cas I-B, CRISPRDirection has ∼30% mismatches, as compared to only ∼4% mismatches for Cas Orientation, which is statistically highly significant (P∼10^–3^). As exceptions, for I-A and I-F, the mismatches are visibly higher for Cas Orientation compared to CRISPRDirection, which will be further discussed below.

As noted in the Introduction, for II-U (i.e., II-C), examples are found in the literature where the *cas* operon and the CRISPR array have the opposite orientation. As seen from Figure 4, such an arrangement appears as a rule, i.e., for this subtype, Cas Orientation exhibits 100% disagreement with the Experimental Orientation. Therefore, Cas Orientation leads to “absolutely inaccurate” predictions, i.e., the method can also be used as a reliable predictor of the CRISPR array direction – with the caveat that for II-U/II-C, the opposite orientation from the *cas* gene direction should be assigned to the CRISPR array. For II-B systems, there are no loci in the Experimental Orientation set – this subtype is small (∼1% of all loci), see Figure 1B, but highly relevant from the point of CRISPR/Cas non-canonical functions (distribution of all loci in the experimental set is provided in Supplementary Figure S2). However, as the small fraction of II-B loci (∼30%), where CRISPRDirection provides predictions perfectly match with Cas Orientation (see Figure 2), we expect that Cas Orientation can reliably predict the array orientation on the II-B set as well.

**FIGURE 5:**
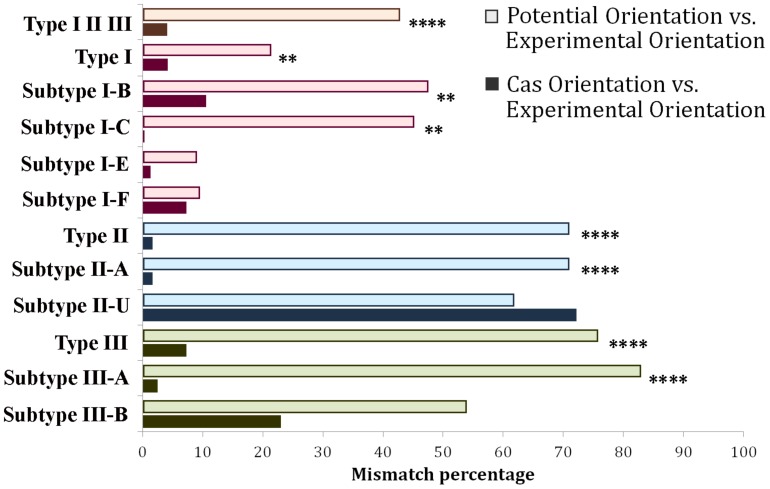
Distribution of mismatches between Potential Orientation vs. ND Experimental Orientation Set (lighter shade bar plots) and Cas Orientation vs. ND Experimental Orientation Set (darker shade bar plots) across different CRISPR/Cas types and subtypes. Every CRISPR/Cas type (along with corresponding subtypes) was denoted with a different color, red corresponding to Type I, blue to Type II, and green to Type III systems. The entire dataset (Type I, Type II, and Type III) was denoted with an orange bar plot. (^∗^*P* < 0.05; ^∗∗^*P* < 0.01; ^∗∗∗^*P* < 0.001; ^∗∗∗∗^*P* < 0.0001). Subtypes I-A, I-D, and II-B were omitted from the figure, due to the very small pool of analyzed arrays (5, 1, and 0, respectively). Note that II-U in the figure (labeled in accordance with CRISPRCasFinder notation), in fact corresponds to the subtype II-C.

Cas Orientation and Potential Orientation are natural competitors in terms of providing predictions for those loci where CRISPRDirection leads to ND assignments. Consequently, in Figure 5 we compare how Cas Orientation and Potential Orientation agree with ND Orientation Set, across all CRISPR/Cas subtypes. These results are in a full agreement with Figure 3C, where we obtained a much higher accuracy of Cas Orientation compared to Potential Orientation on ND Orientation Set. From Figure 5, we see that such result is robustly obtained across almost all CRISPR/Cas subtypes, where a much higher accuracy (which is statistically highly significant) of Cas Orientation is obtained. The exceptions are only I-E and I-F systems, where Cas Orientation still has lower (though not statistically significant) mismatches (Supplementary Table S4).

Consequently, for the arrays where CRISPRDirection provides predictions and for all subtypes but I-A and I-F, Cas Orientation has comparable accuracy to CRISPRDirection. On the large CRISPRDirection ND set, Cas Orientation is clearly better (i.e., leads to a much higher accuracy) than Potential Orientation. Therefore, we propose that Cas Orientation should be used as the method of choice for all CRISPR subtypes (with the exception of I-A and possibly I-F as well), and on the entire dataset (whether or not CRISPRDirection provides prediction). Potential Orientation may be a method of choice for the subtype I-A, though for this subtype, the CRISPRDirection ND set is too small to make reliable (statistically significant) predictions. It is plausible that Cas Orientation is less accurate for the subtype I-A, as the opposite orientation of the CRISPR array and the *cas* genes were documented for this subtype (Garrett et al., 2011; Gudbergsdottir et al., 2011; Lintner et al., 2011; Mousaei et al., 2016; Rollie et al., 2017). Regarding I-F, CRISPRDirection predictor combined with Potential Orientation for ND set overall leads to somewhat better performance compared to Cas Orientation alone. However, even in this case, one might still argue in favor of using a novel method, Cas Orientation, due to its simplicity and straightforward application compared to the combination of CRISPRDirection and Potential Orientation.

### Further Application and Extension of the Analysis

Our method applies to the classified CRISPR arrays (those with a nearby *cas* operon), which are directly associated with effector Cas nucleases. Additionally, there are also a number of orphan CRISPR arrays (arrays without nearby *cas* genes), some of which were found to be functional (e.g., in preventing uptake of active CRISPR/Cas systems) (Almendros et al., 2016). Other orphan arrays are observed to be expressed, but not processed, likely being remnants of previously functional CRISPR/Cas systems (Mandin et al., 2007; Makarova et al., 2015). Also, not all expressed orphan CRISPR arrays can trigger successful interference (Maier et al., 2013), while some detected orphan arrays were subsequently classified as false predictions (Zhang and Ye, 2017). Nevertheless, predicting direction of orphan arrays can be useful, as understanding their physiological roles is still in the beginning, so finding their accurate orientation would be useful. Since Cas Orientation cannot be applied in such cases, CRISPRDirection should be used instead.

Predicting the array orientation also gets more complicated for the bidirectional CRISPR/Cas arrays, i.e., those arrays that can be transcribed in both directions (Charpentier et al., 2015). Currently, none of the three methods assessed here accounts for bidirectional arrays, i.e., they all provide a single (unique) prediction of the array direction, or do not find a prediction at all (for CRISPRDirection and Potential Orientation). However, detecting such cases, by further developing the prediction methods, may be useful to allow better understanding of the functional role of anti-crRNAs (e.g., their role in reducing abundance of crRNAs) (Lillestøl et al., 2009; Richter et al., 2012; Zoephel and Randau, 2013). On the other hand, the cases of bidirectional transcription appear relatively rare (Richter et al., 2012), and have not been (to our knowledge), associated with non-canonical functions up to now (Lillestøl et al., 2009; Richter et al., 2012; Zoephel and Randau, 2013).

Another special case concerns the nested CRISPR arrays, i.e., those arrays where *cas* genes are in-between the two CRISPR arrays. In the case that such arrays are of opposite direction, our method would necessarily lead to a wrong prediction for one of them – that is, it would assign the same direction to both arrays, which is the same as the direction of *cas* genes. However, such prediction errors for Cas Orientation are already accounted for in the presented results, i.e., even with those, our method has about the same accuracy as CRISPRDirection when it provides predictions, and is much more (for almost an order of magnitude) accurate than Potential Orientation when CRISPRDirection does not provide predictions.

Regarding further comparison with experiments, we extensively tested all three methods on available experimental data, and across diverse CRISPR/Cas subtypes. However, further experimental tests would be useful, in particular in those cases where CRISPRDirection leads to ND predictions, while Cas Orientation and Potential Orientation assign different CRISPR array directionality. Another verification of the usefulness of this method would be to utilize it to predict and verify new cases of non-canonical CRISPR/Cas functions. Investigating Type II-B systems may be particularly useful with this respect, as CRISPRDirection leads to a large number of ND assignments in this case, while non-canonical functions in this subtype are well established.

### Summary and Outlook

The direction of the CRISPR array is crucial for the unambiguous prediction of endogenous targets, which is particularly important for large-scale investigations of CRISPR/Cas non-canonical functions. With this goal in mind, we here proposed a novel method Cas Orientation, which provides CRISPR direction prediction for any CRISPR/Cas system, allowing for the analysis not to be restricted to those loci where CRISPRDirection assigns the array orientation. Otherwise, many interesting cases (e.g., 70% of all loci in the highly relevant II-B case) may have to be excluded from the analysis. The method is simple, robust and straightforward to implement, as determining the direction of *cas* genes is close to trivial, e.g., the *cas* gene orientation is readily provided by CRISPRCasFinder. The method does not require any parameterization, in contrast to CRISPRDirection, which employs six different heterogeneous predictors. We showed that Cas Orientation has a high (and robust) accuracy of ∼95% over the entire set of CRISPR/Cas loci; in comparison, the number of mismatches by Potential Orientation increases for a factor of three between CRISPRDirection “non-ND” and “ND” sets – becoming as high as >40% on ND set, where providing accurate predictions is most relevant. Consequently, Cas Orientation may provide an important contribution to a more accurate and straightforward computational analysis of non-canonical CRISPR/Cas functions and CRISPR/Cas systems in general.

Intuitively, codirectionality of the CRISPR array and the *cas* genes observed here, appears consistent with the current assumptions on the CRISPR/Cas evolution. It has been proposed that some of Cas proteins, and the prototype CRISPR repeats, originate from the ancestral Casposone (a self replicating transposone) (Koonin et al., 2017). Accordingly, they initially might had been transcribed together, so the observed dominantly same direction of the CRISPR array and the *cas* genes might be a relic of this. Expression of the *cas* genes and the CRISPR array from the same promoters would have also optimized the interference step through co-regulation at the transcriptional level. As the system architecture diversified to different types and subtypes, and the CRISPR/Cas systems adopted to potentially new roles, the need to re-optimize system functioning lead to novel regulatory patterns. On the other hand, codirectionality of the *cas* genes and the CRISPR array is evidently not a hardwired rule, as in subtype II-C we found it is exactly the opposite, i.e., the *cas* genes and the CRISPR array have opposite orientation. In any case, the rule obtained here might help in shedding light on how CRISPR/Cas made a transition from mobile genetic elements to an adaptive immune system, in addition to providing a novel method for predicting the CRISPR array orientation.

## Data Availability

All datasets generated for this study are included in the manuscript and/or the Supplementary Files.

## Author Contributions

MarD conceived and supervised the work. OM did the large scale data analysis and processing with the help of MarD. JP and BB performed the statistical analysis with the help of MarD and MagD and made the figures with the help of MagD. All authors interpreted the results and wrote the manuscript.

## Conflict of Interest Statement

The authors declare that the research was conducted in the absence of any commercial or financial relationships that could be construed as a potential conflict of interest.
