# Supplementary material for: A Simple Criterion for Inferring CRISPR Array Direction
Source: Front Microbiol. 2019 Sep 4;10:2054. doi: 10.3389/fmicb.2019.02054 (PMC6737040; doi:10.3389/fmicb.2019.02054)
Supplement: Supplementary file 1 [file Data_Sheet_1.PDF]

Supplementary Material

Supplementary Table 1. Percentage of CRISPR loci with non-defined (ND) orientation obtained from CRISPRDirection and Potential Orientation predictors.

CRISPR/Cas (sub)type	%_ND_CRISPRDirection	%_ND_Potential_Orientation	Number of CRISPR loci	Number of CRISPR loci without ND arrays
Type I II III	36	6	5683	3455
Type I	33	5	4626	2925
Subtype I–A	28	4	121	84
Subtype I–B	35	9	647	377
Subtype I–C	31	10	706	430
Subtype I–D	36	11	92	52
Subtype I–E	38	3	2321	1403
Subtype I–F	18	3	739	579
Type II	59	11	533	214
Subtype II–A	58	12	495	203
Subtype II–B	71	0	38	11
Subtype II–U*	45	7	400	206
Type III	38	7	524	316
Subtype III–A	30	8	354	237
Subtype III–B	54	4	170	79

* This subtype was analyzed separately from all other CRISPR/Cas Types and Subtypes.

Supplementary Table 2. Percentage of mismatching results between different CRISPR array orientation predictors. Statistical significance (P value) for difference between CRISPRDirection *vs.* Cas Orientation and Potential Orientation *vs.* Cas Orientation is presented. Statistical significance (P_1 value) for difference between CRISPRDirection *vs.* Cas Orientation and CRISPRDirection *vs.* Potential Orientation is presented.

CRISPR/Cas (sub)type	CRISPRDirection *vs.* Cas Orientation	Potential Orientation *vs.* Cas Orientation	CRISPRDirection *vs.* Potential Orientation	P value	P_1 value
Type I II III	13	23	21	10^{-6}	0.0003
Type I	14	22	19	0.0002	0.01
Subtype I–A	49	60	42	0.2	0.3
Subtype I–B	39	45	49	0.1	0.03
Subtype I–C	7	41	39	10^{-8}	10^{-8}
Subtype I–D	23	54	69	0.02	0.0007
Subtype I–E	4	3	5	0.4	0.4
Subtype I–F	20	31	11	0.02	0.04
Type II	12	30	33	0.02	0.006
Subtype II–A	12	26	30	0.06	0.03
Subtype II–B	0	100	100	0.0005	0.0005
Subtype II–U*	65	21	75	10^{-9}	0.04
Type III	10	29	29	0.005	0.005
Subtype III–A	4	25	23	0.006	0.01
Subtype III–B	29	39	46	0.2	0.1

* This subtype was analyzed separately from all other CRISPR/Cas Types and Subtypes.

Supplementary Table 3. Percentage of mismatching results between different CRISPR array orientation predictors and predetermined Experimental Orientation. Statistical significance (P value) for difference between corresponding percentage categories was given as follows: P_1 – CRISPRDirection mismatch *vs.* Cas Orientation mismatch; P_2 – CRISPRDirection mismatch *vs.* Potential Orientation mismatch; P_3 – Cas Orientation mismatch *vs.* Potential Orientation mismatch. Experimental Pool represents the number of CRISPR loci with both predicted and experimentally determined orientation (from all three predictors).

CRISPR/Cas (sub)type	CRISPRDirection *vs.* Experimental Orientation	Cas Orientation *vs.* Experimental Orientation	Potential Orientation *vs.* Experimental Orientation	P_1 value	P_2 value	P_3 value	Experimental Pool
Type I II III	4	6	14	0.2	0.0004	0.004	2301
Type I	3	6	13	0.2	0.001	0.01	2152
Subtype I–A	34	60	6	0.1	0.09	0.003	35
Subtype I–B	30	4	50	0.003	0.009	10^{-7}	174
Subtype I–C	1	0.5	60	0.5	10^{-12}	10^{-12}	190
Subtype I–D	17	17	50	0.5	0.2	0.2	6
Subtype I–E	0.6	0. 1	0.5	0.5	0.5	0.5	1227
Subtype I–F	0	20	11	0.0004	0.03	0.1	520
Type II	18	0	18	0.1	0.5	0.1	96
Subtype II–A	18	0	18	0.1	0.5	0.1	96
Subtype II–B	/	/	/	/	/	/	0
Subtype II–U*	12	100	91	10^{-28}	10^{-21}	0.0003	138
Type III	15	26	57	0.3	0.004	0.02	53
Subtype III–A	26	26	68	0.5	0.01	0.01	31
Subtype III–B	0	27	41	0.2	0.06	0.3	22

* This subtype was analyzed separately from all other CRISPR/Cas Types and Subtypes.

Supplementary Table 4. Percentage of mismatching results between different Cas Orientation and predetermined Experimental Orientation, and Potential Orientation and predetermined Experimental Orientation. Statistical significance (P value) for difference between corresponding percentages. Experimental Pool represents the number of CRISPR loci with ND-orientation given by CRISPRDirection.

CRISPR/Cas (sub)type	Cas Orientation *vs.* Experimental Orientation	Potential Orientation *vs.* Experimental Orientation	P value	CRISPRDirection ND Experimental Pool
Type I II III	4	43	10^{-14}	600
Type I	5	22	0.007	351
Subtype I–A	20	60	0.2	5
Subtype I–B	11	49	0.008	55
Subtype I–C	0	45	0.003	55
Subtype I–D	100	0	0.2	1
Subtype I–E	1	9	0.2	155
Subtype I–F	8	10	0.4	80
Type II	2	71	10^{-17}	194
Subtype II–A	2	71	10^{-17}	194
Subtype II–B	/	/	/	/
Subtype II–U*	73	62	0.2	37
Type III	7	76	10^{-6}	55
Subtype III–A	2	83	10^{-7}	42
Subtype III–B	23	54	0.2	13

* This subtype was analyzed separately from all other CRISPR/Cas Types and Subtypes.

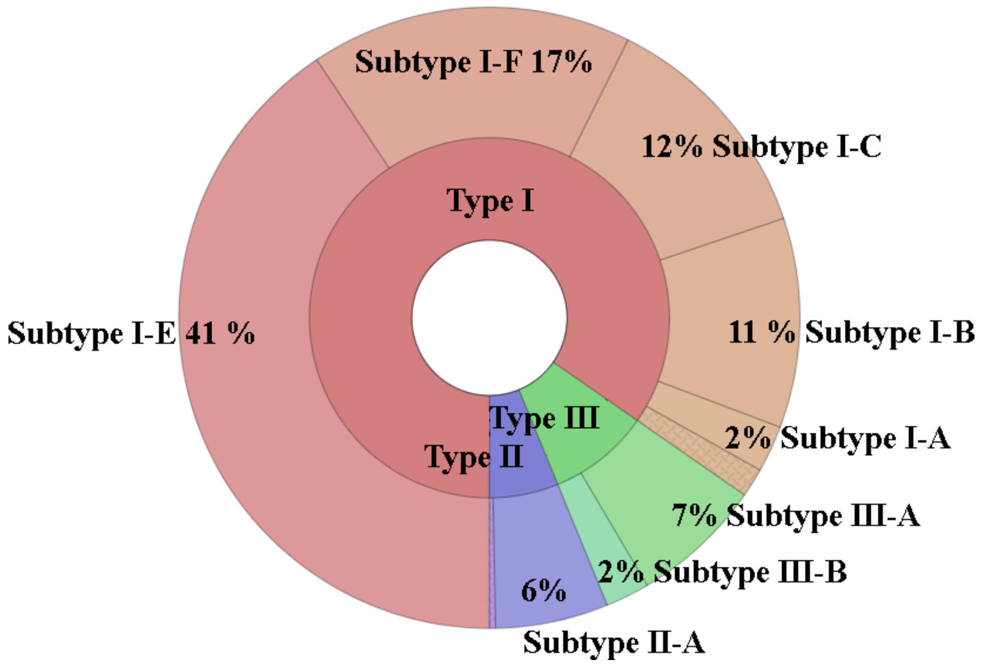

Figure S1. Distribution of CRISPR loci throughout the domains of Bacteria and Archaea, after excluding arrays with ND orientation.

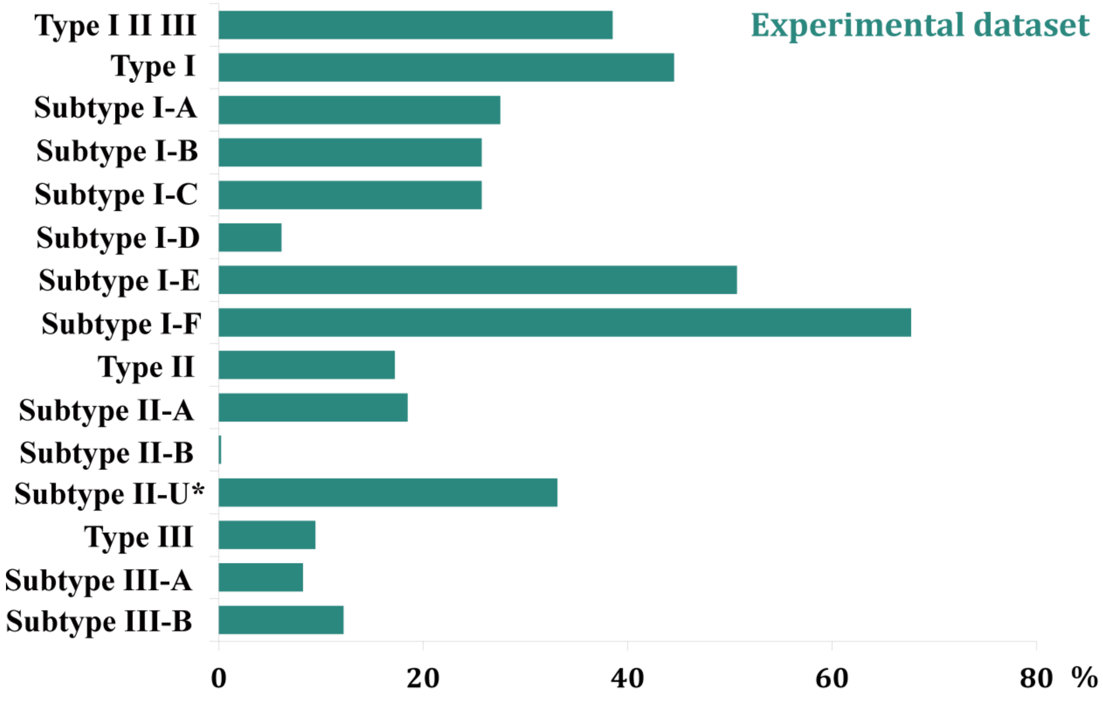

Figure S2. Percent distribution of experimental dataset within all analyzed CRISPR/Cas loci, across different CRISPR/Cas types and subtypes.
